# Perceptions of students and educators regarding a once-off pre-clinical ICU simulation activity

**DOI:** 10.4102/sajp.v78i1.1830

**Published:** 2022-11-21

**Authors:** Ronel Roos, Heleen van Aswegen, Daleen Casteleijn, Catherine H. Thurling

**Affiliations:** 1 Department of Physiotherapy, Faculty of Health Sciences, University of the Witwatersrand, Johannesburg, South Africa; 2 Department of Occupational Therapy, Faculty of Health Sciences, University of the Witwatersrand, Johannesburg, South Africa; 3 Centre for Health Sciences Education, Faculty of Health Sciences, University of Witwatersrand, Johannesburg, South Africa

**Keywords:** cardiopulmonary, clinical practice, high-fidelity, intensive care, physiotherapy, simulation-based education (SBE)

## Abstract

**Background:**

Physiotherapy skills such as suction and manual hyperinflation (MHI) are used to manage patients in intensive care. Performing these skills effectively and safely requires a level of expertise. It is unknown whether a once-off preclinical high-fidelity simulation activity incorporating these skills would translate to clinical practice inclusion.

**Objectives:**

To determine students’ perceptions of a simulation-based education (SBE) activity and clinical educators’ opinions of students’ implementation of skills into practice.

**Method:**

Our study consisted of two parts: a retrospective record review of students’ feedback with the Simulation Effectiveness Tool – Modified (SET-M) and the Simulation Laboratory Questionnaire. A nominal group technique (NGT) with clinical educators provided information on students’ skills implementation. Descriptive data analysis was undertaken.

**Results:**

Six SBE sessions, lasting 3 hours each, with 49 students (*n* = 8–9 students per session) were undertaken. Students perceived the teaching activity positively. Five (33.33%) of 15 clinical educators participated in the NGT. Participants had a mean age of 35.8 (± 8.9) years, were qualified for 13.9 (± 8.9) years and had been supervising students for 7.8 (± 6.7) years. The clinical educators’ top five opinions regarding students’ implementation of the intensive care unit (ICU) skills were: handling skills improved, students had greater confidence performing these skills, students were more observant of a patient’s response to the skill being performed, students had better theoretical knowledge and students had more accurate recall for precautions.

**Conclusion:**

Clinical educators reported a change in students’ clinical practice with regard to skills implementation.

**Clinical implications:**

A once-off preclinical SBE activity influences students’ ICU practice.

## Introduction

Simulation-based education (SBE) is used in health sciences education as a means of optimising and complementing clinical education and can be defined as ‘a set of tools, devices and an environment that mimics an aspect of clinical care’ (Lame & Dixon-Woods 2018). Simulation-based education allows the student to practise a skill and to gain confidence and competency in a safe, controlled environment that replicates the clinical area (Hough et al. 2019). This learning results in the acquisition of knowledge of a skill, competency when performing said skill, improved attitude towards clinical practice and an increase in students’ confidence (Johannessen et al. 2013; Lateef 2010; Mori, Carnahan & Herold 2015). All this occurs while protecting patients from unnecessary risks and harm, as simulators are used (Escudero, Silva & Corvetto 2019). The focus during a clinical simulation is the students, allowing repetitive practice and optimisation of clinical decision-making. During clinical rotations, this is not always possible because of the challenges of clinical placement sites in coping with increased student numbers, resulting in not all students receiving the same clinical exposures. Chetty et al. (2018) observed that staff shortages, lack of equipment and time constraints can negatively influence a student’s work-based clinical skills learning. As mentioned, simulation mimics or replicates reality, and fidelity is the term that refers to the realism of the equipment or simulator and the environment to enable students to suspend reality and immerse themselves in the simulated experience (Al-Elq 2010). High-fidelity simulators are full-body computerised mannikins that provide a close-to-reality experience producing physiological responses to procedures performed (Mossoth et al. 2019). Educators can use this technology to simulate a ‘real-world’ clinical case, during which students can refine their practice through facilitation in a supportive environment (Al-Elq 2010; Blackstock & Jull 2007).

Minimal clinical standards for entry-level physiotherapists to work in intensive care units (ICUs) are suggested to include integrated medical knowledge, a multidisciplinary team approach to patient management and physiotherapy practice content (Van Aswegen et al. 2017). High-fidelity simulation is a positive learning opportunity to introduce students to the ICU environment to increase their interest in working in the area and lessen discomfort with working in the high-risk environment (Mori et al. 2015). Assessment of physiotherapy student performance regarding the following skills is also possible with a high-fidelity simulator: assessing bed mobility and respiratory status of the ‘patient’, communication with the ‘patient’ and recognising clinical status change and responding to change (Mori et al. 2015). Ohtake et al. (2013) observed students’ improved confidence with technical skills and clinical reasoning, and most (98%) reported the simulation experience to be beneficial. Three additional studies included in the systematic review by Mori et al. (2015) indicated that exposing students to high-fidelity clinical cases reduces students’ anxiety regarding working in ICU and improves students’ confidence when managing a patient in ICU, and students report high satisfaction with the learning experience.

Physiotherapists working with patients with respiratory impairments often need to assist patients to remove excess tracheobronchial secretions if patients are unable to cough effectively and clear their secretions independently (Ntoumenopoulus et al. 2017). Airway suction is a modality that is used to care for such a patient (Branson, Gomaa & Rodriquez 2014). Manual hyperinflation (MHI) is a technique during which a patient is disconnected from a mechanical ventilator and given manual breaths with a reservoir bag-valve circuit to provide a breath larger than the patient’s tidal volume or ventilator set tidal volume, if the patient is not spontaneously breathing (Ntoumenopoulus et al. 2017; Van Aswegen et al. 2017). The technique is beneficial in improving a patient’s lung compliance and oxygenation levels and increasing sputum clearance (Paulus et al. 2012). Knowledge, understanding and competency executing suction and MHI are required to manage an ICU patient safely and effectively, thereby complying with minimal standards of practice for entry-level physiotherapists (Hanekom et al. 2015; Van Aswegen et al. 2017). Less is known whether high-fidelity simulation of suction and MHI skills before initiation of students’ clinical ICU rotations would translate to inclusion of such skills in their clinical practice. The aim of our study was therefore to determine students’ perceptions of a high-fidelity simulation activity of suction and MHI skills training and their clinical educators’ opinions on whether students’ implementation of these skills during clinical practice changed following the once-off SBE activity.

## Methods

Prior to 2017, the training that undergraduate physiotherapy students at the University of the Witwatersrand received to prepare them for working in an ICU setting consisted of theoretical classes to deliver the curriculum content and one compulsory 4-week clinical rotation in ICU. The SBE project was undertaken in 2017–2018. The project consisted of two parts: Firstly, a retrospective record review of undergraduate final-year physiotherapy students’ feedback of a once-off preclinical high-fidelity ICU simulation experience performed in 2017 using the Simulation Effectiveness Tool – Modified (SET-M) questionnaire and the university’s Simulation Laboratory Questionnaire. Secondly, a nominal group technique (NGT) with the clinical educators who supervised the students during their ICU clinical rotation in 2017 was undertaken to provide information on translation of skills into clinical practice.

Teaching of the ICU skills was adapted in 2017 to include SBE in the university’s new simulation laboratory. The SBE sessions were held on the first day of the students’ ICU rotation prior to them being involved in clinical patient care. A simulation session started with skills training. The student group, comprising eight to nine students, was divided into two skills groups. Training of each skill lasted ± 45 min (4–5 students per skills group) and consisted of demonstration, practice with educator and peer feedback regarding the skill performance. After completion of the skills training section of the SBE session, the students underwent a prebriefing session to prepare them regarding the different roles and expectations of the high-fidelity simulation. A high-fidelity ICU clinical case simulation followed to further assist with clinical decision-making while managing the patient with suction and MHI. A facilitator-led approach during the simulation was performed to provide a supportive environment. During the high-fidelity clinical case simulation, an array of skills were covered: observational skills, assessment skills, communication (verbal and written) and the execution of suction and MHI.

Following the case simulation, the students provided feedback regarding the simulation activity and experience by completing the SET-M and the institution’s Simulation Laboratory Questionnaire at the end of each session. The SET-M questionnaire consists of questions arranged into four categories to allow participants to give feedback regarding the simulation prebrief, their development of learning, their development of confidence and the debriefing activity following the simulation event (Leighten et al. 2015). Students gave feedback via a Likert scale consisting of the following options, for example, ‘do not agree’, ‘somewhat agree’, ‘strongly agree’ and ‘not applicable’. An online application was submitted for its use at Evaluating Healthcare Simulation (available at: https://sites.google.com/view/evaluatinghealthcaresimulation/set-m?authuser=0). The institution’s Simulation Laboratory Questionnaire consists of eight questions where students provide feedback regarding the simulation activity according to a Likert scale, for example, strongly disagree = 1, neutral = 3 and strongly agree = 5. Both tools were included in our study as the SET-M provided more in-depth feedback regarding the immersive case-based high-fidelity simulation activity compared with the institution’s tool that students completed to provide feedback regarding educators’ teaching, the alignment of the SBE with their curriculum and venue particulars.

At the end of the academic year, clinical educators who supervised the students during their ICU clinical rotation were invited to participate in the NGT. The focus group was held in a quiet venue in the Department of Physiotherapy at the University of the Witwatersrand. Participants completed a questionnaire at the start of the session to provide information on their demographics and clinical experience. The NGT then followed. Nominal group technique is a consensus method used to solve problems, generate ideas and determine priorities (McMillan, King & Tully 2016). The steps taken during the NGT were guided by McMillan et al. (2016). The facilitator, a senior academic and author not employed in the physiotherapy department, asked participants to reflect on the final-year students’ implementation of suction and MHI skills during patient care. Participants silently reflected on the topic; each participant then shared his or her ideas in a round robin by using sticky notes until no more ideas were generated. Step three was a discussion of ideas, grouping of similar ideas and exclusion of redundant suggestions. During step four, participants selected five ideas and ranked them in the order of preference, with the highest ranking receiving a score of five and the lowest rank a score of one. During the session, the last author kept notations on a laptop to ensure that the ideas shared were not lost. Ideas from the sticky notes were captured in a Microsoft Excel spreadsheet (Microsoft Corporation, Redmond, Washington, United States). After completion of the session, content from the sticky notes was compared with the Microsoft Excel spreadsheet to ensure that all information was captured. Finally, the scores of each selected idea or opinion were summed and presented to the group for discussion. As a result of time constraints, reranking occurred electronically, with participants completing a Research Electronic Data Capture (REDCap; Vanderbilt University, Nashville, Tennessee, United States) survey hosted at the tertiary institution. This was carried out to finalise the listed ideas and opinions and to select items discussed during the NGT that participants considered barriers to students’ implementing the skills into practice.

Students’ feedback with the respective questionnaires was analysed with descriptive analysis (frequencies and percentages) and is presented as tables. Clinical educators’ demographics and clinical experience information were analysed with descriptive analysis. Analyses were performed with the Statistical Package for the Social Sciences (SPSS; IBM Corporation, Armonk, New York, United States), and the Shapiro–Wilk test was used to evaluate normality of data.

### Ethical considerations

Ethical clearance to conduct our study was obtained from the University of the Witwatersrand Human Research Ethics Committee (reference number: M180151). Permission from the head of the Physiotherapy Department and head of the Centre for Health Sciences Education of the tertiary institution was received to access the students’ feedback as per the two respective questionnaires. The permissions of the heads of the Physiotherapy Department and the respective clinical sites were gained to contact the relevant clinical educators for potential participation in the NGT. Nominal group technique participants consented in writing to participate in the study and allowed voice recording of the group discussions.

## Results

Six SBE sessions were held in the 2017 academic year, lasting 3 hours each, with a total of 49 students (*n* = 8–9 students per session). Thirty-three students (71.8%) completed the institution’s Simulation Laboratory Questionnaire (Table 1). Two students did not complete the last three questions of the questionnaire, and their results were excluded from the analysis.

**TABLE 1:** Students’ feedback as per the Simulation Laboratory Questionnaire (*n* = 31).

Statement on questionnaire	Neutral		Agree		Strongly agree	
	*n*	%	*n*	%	*n*	%
The simulation activity was aligned to your curriculum and course objectives.	-	-	2	6.5	29	93.5
You were provided with clear learning objectives prior to the simulation activity taking place (preparation).	-	-	4	12.9	27	87.1
The simulation activity promoted learning that can be integrated into your clinical practice (relevancy).	-	-	0	0.0	31	100.0
The simulation teaching and learning approach was relevant to your level of study and promotes learning and understanding in the subject.	-	-	1	3.2	30	96.8
The simulation equipment was contextually appropriate and provided a realistic clinical environment.	-	-	5	16.1	26	83.9
The simulation space provided physical and psychological safety for you as a student.	-	-	7	22.6	24	77.4
The simulation facilitator provided support and feedback to students during the simulation activity.	-	-	2	6.5	29	93.5
The simulation and clinical staff have the relevant clinical knowledge, understand the specific learning objectives and possess the required clinical teaching skills to facilitate the theory practice.	-	-	1	3.2	30	96.8

All students (*n* = 31, 100%) felt that the simulation activity promoted learning and clinical relevance. In addition, 30 (96.8%) students felt that the teaching and learning approach was relevant to their level of study and promoted learning and understanding of the subject covered.

Forty-five SET-M questionnaires (91.9%) were completed, but six were excluded because of incomplete data (Table 2). Feedback from students were mostly in the ‘strongly agree’ category of the SET-M questionnaire.

**TABLE 2:** Students’ feedback as per the Simulation Effectiveness Tool – Modified questionnaire (*n* = 39).

Statement on questionnaire	Somewhat agree		Strongly agree		Not applicable	
	*n*	%	*n*	%	*n*	%
Prebriefing increased my confidence.	-	-	39	100.0	-	-
Prebriefing was beneficial to my learning.	-	-	39	10.0	-	-
I am better prepared to respond to changes in my patient’s condition.	2	5.1	37	94.9	-	-
I developed a better understanding of the pathophysiology.	3	7.7	36	92.3	-	-
I am more confident of my patient assessment skills.	2	5.1	37	94.9	-	-
I had an opportunity to practise my clinical decision-making skills.	1	2.6	37	94.9	1	2.6
I felt empowered to make clinical decisions.	3	7.7	36	92.3	-	-
I am more confident in my ability to prioritise care and interventions.	1	2.6	38	97.4	-	-
I am more confident in communicating with my patient.	2	5.1	37	94.9	-	-
I am more confident in my ability to teach patients about their illness and interventions.	4	10.3	35	89.7	-	-
I am more confident in my ability to report information to the healthcare team.	1	2.6	38	97.4	-	-
I am more confident in providing interventions that foster patient safety.	1	2.6	38	97.4	-	-
I am more confident in using evidence-based practice in my domain of healthcare.	1	2.6	38	97.4	-	-
Debriefing allowed me to verbalise my feelings before focusing on the scenario.	-	-	39	100.0	-	-
Debriefing allowed opportunities to self-reflect on my performance during simulation.	-	-	39	100.0	-	-
Debriefing was valuable in helping me improve my clinical judgement.	-	-	39	100.0	-	-
Debriefing was a constructive evaluation of the simulation.	-	-	39	100.0	-	-
Debriefing contributed to my learning.	-	-	39	100.0	-	-

*Source:* Statements on questionnaire adapted from Leighton, K., Ravert, P., Mudra, V. & Macintosh, C., 2018, *Simulation Effectiveness Tool - Modified*, viewed n.d. from https://sites.google.com/view/evaluatinghealthcaresimulation/set-m

Fifteen clinical educators were invited to participate in the NGT, and five (33.3%) consented. The educators had a mean age of 35.8 (± 8.9) years; all were female (*n* = 5, 100%), qualified for 13.9 (± 8.9) years and had been clinical educators and supervising students for 7.8 (± 6.7) years.

The clinical educators outlined barriers that may influence students’ implementation of skills into clinical practice, and these barriers could be multifactorial (Table 3 and Table 4).

**TABLE 3:** Final ranking of clinical educators’ opinions on skills implementation during intensive care unit clinical practice.

Description	Total score	Rank
Handling techniques improved in both skills.	24	1
Greater confidence to perform skills on patients.	23	2
More observant of patient’s response to skill performed.	22	3
Better theoretical knowledge for both techniques.	22	3
Recall of precautions for both skills more accurate.	21	4
Students are more willing to try both suction and MHI.	20	5
Selected correct equipment to perform both skills.	20	5
Students are more eager to perform MHI.	17	6
Students are more aware of infection control with suction.	17	6

MHI, manual hyperinflation.

**TABLE 4:** Barriers to skills implementation into clinical practice.

Barriers
Fear of real patients and real scenarios
Suction: open versus closed. Opportunity to practise open versus closed is facility dependent.
Simulation training is not always carried over into clinical practice because of a lack of equipment at some clinical placements.
Simulation training does not always carry over to clinical practice because of the culture at a unit regarding MHI.
Students’ confidence affected by facilities and equipment available to them.
Experience and confidence of supervisor affects learning outcomes of students.
A negative experience using this technique as a treatment modality on an acute care patient.

MHI, manual hyperinflation.

## Discussion

Students found the high-fidelity SBE experience positive, with all perceiving the activity as clinically relevant and most reporting the prebriefing and debriefing to be beneficial to their learning. The simulation session was planned at the beginning of their ICU rotation as a means for greater translation of skills teaching into clinical practice. Clinical educators observed improvement in translation of skills into clinical practice following the once-off high-fidelity SBE activity during the students’ clinical rotation. The clinical educators reported that students’ knowledge of said techniques improved in addition to their practical ability to execute the skills safely in the ICU environment. This finding is important considering that the theoretical teaching related to the ICU skills was not adjusted in the undergraduate curriculum during the time of our study. In addition, it should, however, be observed that mentoring in the provision of these techniques from their peers and clinical educators during their rotations may have also played a role.

The SBE session was created in such a way that students had a period of deliberate practice of suction and MHI separately, followed by the high-fidelity ICU clinical case simulation where skills were combined. During the high-fidelity clinical case simulation, the following skills were worked on: students communicated with the ICU patient on the treatment to be performed; they conducted assessment skills, for example, evaluated and interpreted the vital signs from the ICU monitor and performed chest wall auscultation of the patient; screening of the precautions related to the pertinent ICU skills followed; they then executed the skills and observed the effect of techniques on the patient; outcome measures were re-evaluated following skills performance; and lastly, they documented their treatment. An array of clinical skills was thus covered with one clinical case scenario.

The benefits of SBE reported in the literature support our study’s findings that the confidence of students improved when performing said skills in the clinical domain. Mori et al. (2015), when conducting a systematic review of the literature related to SBE in undergraduate physiotherapy curricula, observed that SBE was well received by students; it assisted with skills attainment and helped adjust students’ behaviour. In addition, Hough et al. (2019) indicate that SBE had a significant influence on undergraduate physiotherapy students’ self-efficacy when performing assessment skills and managing paediatric patients. Mansell, Harvey and Thomas (2020) evaluated the effect of adding SBE to an on-call training programme for qualified physiotherapists who had a nonrespiratory speciality background and were required to do on-call service at a hospital. The authors found that the addition of SBE to the on-call training programme improved physiotherapists’ self-confidence from pre- to post-training, and this was highly beneficial as participants often found performing an on-call duty a very stressful experience (Mansell et al. 2020).

The clinical educators highlighted barriers that could influence students’ translation of skills into clinical practice. Barriers included lack of equipment at some clinical placements, the influence of the clinical supervisor’s experience as it relates to said skills in the clinical domain and the student’s fear of the ICU patient and real-life scenario. Chetty et al. (2018) observed that a lack of equipment can negatively influence a student’s work-based clinical skills learning. In addition, the authors reported that the student–educator relationship and clinical personal collaboration were vital to ensure carry-over of education from the classroom to the healthcare setting in a South African context. It is difficult to influence a lack of equipment at a healthcare setting, as one reason for this barrier could be because of a lack of finances in low-resource settings; however, suction and MHI equipment are well-recognised basic life support tools used in the resuscitation of patients in ICU; hence, one would expect to find such equipment in all ICUs of clinical settings. Implementing education programmes and SBE for clinical educators could be a way to influence some of the barriers that were found.

Undergraduate physiotherapy students often feel overwhelmed by the ICU environment and lack confidence in performing clinical skills on patients who are dependent on mechanical ventilation, despite having theoretical preparation and exposure to the ICU clinical environment before their clinical rotation (Major et al. 2020). It is recommended that undergraduate physiotherapy curricula should include authentic learning experiences to optimally prepare students for their profession (Major et al. 2020). Our study’s single-session SBE training was an attempt to decrease students’ fears for the clinical ICU setting. The students all reported that the SBE session improved their learning, and the clinician participants felt that after SBE, the students had better handling skills when performing suction and MHI and greater confidence in performing these techniques on patients. This single-session preclinical SBE experience created the opportunity for the students to learn on the two highest levels of Miller’s pyramid of clinical competence (the ‘shows how’ and the ‘does’) in a nonthreatening environment and can be seen as successful in better preparing the students to perform suction and MHI skills on patients in the ICU (Major et al. 2020).

The cost related to SBE, in terms of time dedicated to the activity by physiotherapy academic staff and simulation laboratory technical staff and equipment costs, needs consideration and future study. Typically, SBE in physiotherapy education is delivered over more than one session (Hough et al. 2019; Wright et al. 2018). The positive effect of this single-session SBE on students’ learning and levels of confidence suggests that costs could be limited when education is delivered in this fashion.

A limitation is that our study was conducted at a single tertiary institution in South Africa, and findings should be interpreted within this context. An additional limitation was that clinical educators might not have been present each time when students performed suction and MHI on a patient. Therefore, they may have only observed successful application of these skills when accompanying a student during their clinical rotation. Lastly, the clinical educators were aware of the new SBE activity during 2017, but their NGT was held when the whole student cohort had finished their ICU rotation. Recall bias might therefore have influenced the qualitative data collected.

## Conclusion

High-fidelity simulation training of cardiopulmonary skills in the simulation laboratory is a beneficial adjunct teaching method to the physiotherapy ICU curriculum for final-year students. Simulation integration into the undergraduate curriculum requires a team approach to make it successful, as faculty team members with teaching content experience as well as staff with simulation technology expertise are required for SBE. Clinical educators reported changes in students’ clinical practice during their ICU rotation following SBE; however, implementation of skills into clinical practice may be hampered by context-specific barriers that require adjustment where possible.

## References

[CIT0001] Al-Elq, A.H., 2010, ‘Simulation-based medical teaching and learning’, *Journal of Family & Community Medicine* 17(1), 35–40. 10.4103/1319-1683.68787 22022669 PMC3195067

[CIT0002] Blackstock, F.C. & Jull, G.A., 2007, ‘High fidelity patient simulation in physiotherapy education’, *Australian Journal of Physiotherapy* 53(1), 3–5. 10.1016/S0004-9514(07)70056-9 17326733

[CIT0003] Branson, R.D., Gomaa, D. & Rodriquez, D., 2014, ‘Management of the artificial airway’, *Respiratory Care* 59(6), 974–989. 10.4187/respcare.03246 24891202

[CIT0004] Chetty, V., Maddocks, S., Cobbing, S., Pefile, N., Govender, T., Shah, S.A. et al., 2018, ‘Physiotherapy clinical education at a South African university’, *African Journal of Health Professions Education* 10(1), 1–6. 10.7196/AJHPE.2018.v10i1.987

[CIT0005] Escudero, E., Silva, M. & Corvetto, M., 2019, *Simulation: A training resource for quality care and improving patient safety*, viewed 07 September 2022, from https://www.intechopen.com/chapters/68899.

[CIT0006] Hanekom, S., Van Aswegen, H., Plani, N. & Patman, S., 2015, ‘Developing minimum clinical standards for physiotherapy in South African intensive care units: The nominal group technique in action’, *Journal of Evaluations in Clinical Practice* 21(1), 118–127. 10.1111/jep.12257 25267001

[CIT0007] Hough, J., Levan, D., Steele, M., Kelly, K. & Dalton, M., 2019, ‘Simulation-based education improves student self-efficacy in physiotherapy assessment and management of paediatric patients’, *BMC Medical Education* 19(46), 1–11. 10.1186/s12909-019-1894-2 31842864 PMC6915888

[CIT0008] Johannessen, E., Silen, C., Kvist, J. & Hult, H., 2013, ‘Students’ experiences of learning manual clinical skills through simulation’, *Advances in Health Science Education Theory and Practice* 18(1), 99–114. 10.1007/s10459-012-9358-z 22395307

[CIT0009] Lame, G. & Dixon-Woods, M., 2018, ‘Using clinical simulation to study how to improve quality and safety in healthcare’, *BMJ Simulation & Technology Enhanced Learning* 6(2), 87–94. 10.1136/bmjstel-2018-000370 PMC7056349 32133154

[CIT0010] Lateef, F., 2010, ‘Simulation-based learning: Just like the real thing’, *Journal of Emergency, Trauma and Shock* 3(4), 348–352. 10.4103/0974-2700.70743 PMC2966567 21063557

[CIT0011] Leighten, K., Ravert, P., Mudra, V. & Macintosh, C., 2015, ‘Updating the simulation effectiveness tool: Item modifications and re-evaluation of psychometric properties’, *Nursing Education Perspectives* 36(5), 317–323. 10.5480/15-1671 26521501

[CIT0012] Leighton, K., Ravert, P., Mudra, V. & Macintosh, C., 2018, *Simulation Effectiveness Tool - Modified*, viewed n.d., from https://sites.google.com/view/evaluatinghealthcaresimulation/set-m 10.5480/15-1671 26521501

[CIT0013] Major, M.E., Ramaekers, S.P.J., Engelbert, R.H.H. & Van der Schaaf, M., 2020, ‘Preparing undergraduate students for clinical work in a complex environment: Evaluation of an e-learning module on physiotherapy in the intensive care unit’, *BMC Medical Education* 20(130), 1–10. 10.1186/s12909-020-02035-2 PMC7189528 32345330

[CIT0014] Mansell, S.K., Harvey, A. & Thomas, A., 2020, ‘An exploratory study considering the potential impacts of high-fidelity simulation based education on self-evaluated confidence of non-respiratory physiotherapists providing an on-call respiratory physiotherapy service: A mixed methods study’, *BMJ Simulation & Technology Enhanced Learning* 6(4), 199–205. 10.1136/bmjstel-2019-000444 PMC7399866 32832100

[CIT0015] McMillan, S.S., King, M. & Tully, M.P., 2016, ‘How to use the nominal group and Delphi techniques’, *International Journal of Clinical Pharmacology and Therapeutics* 38, 655–662. 10.1007/S11096-016-0257-X PMC4909789 26846316

[CIT0016] Mori, B., Carnahan, H. & Herold, J., 2015, ‘Use of simulation learning experiences in physical therapy entry-to-practice curricula: A systematic review’, *Physiotherapy Canada* 67(2), 194–202. 10.3138/ptc.2014-40E 25931672 PMC4407134

[CIT0017] Mossoth, C., Roder, H., Ohlenburg, H., Hessler, M., Zarbock, A., Popping, D.M. et al., 2019, ‘High- fidelity is not superior to low fidelity simulation but leads to overconfidence in medical students’, *BMC Medical Education* 19(29), 1–8. 10.1186/s12909-019-1464-7 30665397 PMC6341720

[CIT0018] Ntoumenopoulus, G., Hammond, N. & Watts, N.R., Thompson, K., Hanlon, G., Paratz, J.D. et al., 2017, ‘Secretion clearance strategies in Australian and New Zealand intensive care units’, *Australian Critical Care* 31(4), 191–196. 10.1016/j.aucc.2017.06.002 28662942

[CIT0019] Ohtake, P.J., Lazarus, M., Schillo, R. & Rosen, M., 2013, ‘Simulation experience enhances physical therapist student confidence in managing a patient in the critical care environment’, *Physical Therapy* 93(2), 216–228. 10.2522/ptj.20110463 23329555

[CIT0020] Paulus, F., Binnekade, J.M., Vroom, M.B. & Schultz, M.J., 2012, ‘Benefits and risks of manual hyperinflation in intubated and mechanically ventilated intensive care unit patients: A systematic review’, *Critical Care* 16(4), R145. 10.1186/cc11457 22863373 PMC3580733

[CIT0021] Van Aswegen, H., Patman, S., Plani, N. & Hanekom, S., 2017, ‘Developing minimal clinical standards for physiotherapy in South African ICUs: A qualitative study’, *Journal of Evaluation in Clinical Practice* 23(6), 1258–1265. 10.1111/jep.12774 28548368

[CIT0022] Wright, A., Moss, P., Dennis, D.M., Harrold, V., Levy, S., Furness, A.L. et al., 2018, ‘The influence of a full-time, immersive simulation-based clinical placement on physiotherapy student confidence during the transition to clinical practice’, *Advances in Simulation* 3, 3. 10.1186/s41077-018-0062-9 29484204 PMC5819286

